# National Health Insurance Fund’s relationship to retail drug outlets: a Tanzania case study

**DOI:** 10.1186/s40545-021-00303-0

**Published:** 2021-02-17

**Authors:** Martha Embrey, Romuald Mbwasi, Elizabeth Shekalaghe, Jafary Liana, Suleiman Kimatta, Gasto Ignace, Angel Dillip, Tamara Hafner

**Affiliations:** 1 Management Sciences for Health, Arlington, VA USA; 2 Apotheker Consultancy Co., Ltd, Dar es Salaam, Tanzania; 3 Pharmacy Council of Tanzania, Dodoma, Tanzania; 4 Management Sciences for Health/Medicines, Technologies, and Pharmaceutical Services Program, Arlington, USA

**Keywords:** National health insurance scheme, Retail drug outlet, Pharmaceutical benefits, Accredited drug dispensing outlet, Pharmacy, Tanzania, Tanzania National Health Insurance Fund

## Abstract

**Background:**

Achieving universal health coverage will require robust private sector engagement; however, as many low- and middle-income countries launch prepayment schemes to achieve universal health coverage, few are covering products from retail drug outlets (pharmacies and drug shops). This case study aims to characterize barriers and facilitators related to incorporating retail drug outlets into national prepayment schemes based on the experience of the Tanzanian National Health Insurance Fund’s (NHIF) certification of pharmacies and accredited drug dispensing outlets.

**Methods:**

We reviewed government documents and interviewed 26 key informants including retail outlet owners and dispensers and central and district government authorities representing eight districts overall. Topics included awareness of NHIF in the community, access to medicines, claims processing, reimbursement prices, and how the NHIF/retail outlet linkage could be improved.

**Results:**

Important enablers for NHIF/retail outlet engagement include widespread awareness of NHIF in the community, NHIF’s straightforward certification process, and their reimbursement speed. All of the retail respondents felt that NHIF helps their business and their clients to some degree. As for barriers, retailers thought that NHIF needed to provide more information to them and to its members, particularly regarding coverage changes. Some retailers and government officials thought that the product reimbursement prices were below market and not adjusted often enough, and pharmacy respondents were unhappy about claim rejections for what they felt were insignificant issues. All interviewees agreed that one of the biggest problems is poor prescribing practices in public health facilities. They reiterated that prescribers need more supervision to improve their practices, particularly to ensure adherence to standard treatment guidelines, which NHIF requires for approving a claim. In addition, if a prescription has any problem, including a wrong date or no signature, the client must return to the health facility to get it corrected or pay out-of-pocket, which is burdensome.

**Conclusions:**

Little published information is available on the relationship between health insurance plans and retail providers in low- and middle-income countries. This case study provides insights that countries can use when designing ways to include retail outlets in their health insurance schemes.

## Introduction

To achieve universal health coverage (UHC), many low- and middle-income countries are launching prepayment schemes, such as national health insurance. Not all schemes, however, include pharmaceuticals and other commodities, although the common out-of-pocket spending on this category can easily lead to catastrophic expenditure. At the UN General Assembly’s high-level meeting on UHC in September 2019, government leaders recognized that inadequate access to health products was one of the barriers to achieving the goal of UHC by 2030 [1]. Moreover, medicines are often purchased out-of-pocket at private sector providers, such as pharmacies and drug shops, which schemes sporadically include in their benefits coverage. Therefore, incorporating not only medicines into health insurance schemes but also medicines from retail outlets seems a promising choice for countries striving for UHC. Countries are recognizing the private sector gap and are moving toward including private sector providers in their national insurance schemes for reasons such as the desire to supplement public-sector capacity and increase quality in the private sector by enforcing standards [2]. The incorporation of retail outlets may not be a top priority for UHC policy makers, but ultimately, achieving UHC will require the thoughtful inclusion of all kinds of private providers in national health insurance schemes, including retail drug outlets [3].

Because few national health insurance schemes in low-income countries cover products from retail drug outlets, little published information is available on the relationship between plans and retail providers. Although health insurance coverage of medicines from retail outlets undoubtedly helps increase access to and affordability of essential medicines by decreasing out-of-pocket spending [4], problems with the arrangement have been perceived from both sides—health insurance programs and the outlets. For example, 12 out of 18 insurance plans in sub-Saharan Africa reported that their most common problem was provider complaints about delays in settling claims, and almost as many reported that fraud was a problem, although the argument could be that the complaints about delays should be on the side of the providers, not from the health insurance plans [5]. In Ghana, studies indicated that the National Health Insurance Authority’s certification process for outlets generally worked well except for delays in claim reimbursement and in the process to sign up retail outlets for certification, which took up to 12 months [3]. In a survey of Kenya’s outlets, on the other hand, participants felt that the unwieldy certification process and lack of knowledge about how the National Health Insurance Fund worked kept providers from participating [3]. Meanwhile, private sector pharmacies in Indonesia were hampered in their procurement efforts because of the prepayment plan’s inefficient e-catalog system [6].

To shed more light on this relationship and to provide information that other countries can consider as they design ways to incorporate retail drug outlets into prepayment schemes, we interviewed informants in Tanzania to characterize the experiences related to the National Health Insurance Fund’s (NHIF) coverage of medicines through pharmacies and accredited drug dispensing outlets (ADDOs). This case study is exploratory and aims to characterize the barriers and facilitators related to incorporating retail drug outlets in national prepayment schemes. Tanzania serves as a suitable context for exploring this topic because of its success with the ADDO program and its established history with prepayment schemes. Tanzania’s NHIF has incorporated pharmacies as providers for more than 20 years, and the fund also covers ADDOs, and yet little has been documented on this relationship. We focused on NHIF for this case study, because the country’s larger prepayment plan, the Community Health Fund (CHF), known now as the improved Community Health Fund (iCHF), does not cover purchases from pharmacies or ADDOs.

## Overview of Tanzania’s Prepayment Schemes

Tanzania instituted user fees in 1993 to supplement health sector resources, build community ownership, and make service providers more accountable. To offset the negative effects of user fees, the country began to roll out prepayment schemes, including the CHF in rural areas, the urban version of CHF, Tiba Kwa Kadi (TIKA), and NHIF for civil servants and some private sector employees (Table 1). The Social Health Insurance Benefit is another scheme that covers a small proportion of private sector workers. Population coverage by the largest prepayment schemes, iCHF and NHIF, has increased over the years to 32% in 2019 (G3). As of 2018, iCHF covered 25% of households, but coverage across 25 of the country’s 31 regions ranged from 4 to 78% [7]. By December 2019, NHIF covered 9% of the population and iCHF had decreased to 23% (G3).

**Table 1:** Summary of Tanzania’s prepayment schemes

Scheme	Improved Community Health Fund (iCHF)	Tiba Kwa Kadi (TIKA)	National Health Insurance Fund (NHIF)	Social Health Insurance Benefit
Year started	1996 (CHF)	2009	1999–2001	2005
Membership	Rural informal sector	Urban informal sector	Civil servants, some private sector	Private sector workers
Annual premium	Voluntary TSH 30,000		Mandatory 3% of salary for civil servants; voluntary vifurushi plans TSH 30,000–100,000	Voluntary 20% of salary
Benefits package	Primary health care and limited hospital care; medicines access at health facility		Inpatient/ outpatient at any certified facility; medicines access at health facility or retail outlet	Similar to NHIF
Population coverage as of 2019	23%		9%	< 1%

According to central-level government informants, the national goal for combined iCHF and NHIF population coverage in 2020 is 50%, up from the current 32%

The government launched an “improved” CHF in 2018 to boost CHF enrollment, and is rolling it out district by district with the aim to increase access to quality healthcare for people in the informal sector, mostly rural and low-income groups. The primary improvement was to make the scheme more portable by expanding service coverage from the one primary health facility, where the member was registered, to multiple public health facilities throughout the region—up to regional referral hospital level. iCHF members were also given priority for services at the facility. Additionally, enrollment officers were assigned to villages to make the enrollment process more proactive. However, the iCHF benefits package is still very basic compared to that of the NHIF. Moreover, some Tanzanians view iCHF coverage as useless, and many members quit their membership due to long distances to facilities where services are covered [8]. Studies looking at the barriers of people enrolling or re-enrolling in CHF and iCHF noted that lack of drugs at health facilities meant buying them out-of-pocket at ADDOs or pharmacies, where they were not covered, at a cost that approached that of the cost of the iCHF premium for some members [8, 9].

Designed for civil servants, NHIF is a mandatory scheme comprising a 6% payroll deduction split between the government and employee. NHIF membership covers the employee, their spouse, and no more than four dependents under 18 years. To increase enrollment, NHIF started opening up membership to anyone in the country on a voluntary basis in 2009. These *vifurushi* (bundles) include a wide range of products for various groups, including informal workers, who are also primary targets for iCHF and TIKA. However, the NHIF views these packages as complementary to iCHF rather than competitive, because they are more expensive and comprehensive; varying vifurushi annual costs include those for students under 18 years (TSH 54,000), farmers (TSH 30,000), and boda (motorcycle taxi) drivers (TSH 100,000), with different levels of benefits (G1). More than 67% of the membership is still comprised of public employees [7]. Despite maintaining substantial reserves, sustainability has been an issue for NHIF as it has seen a significant rise in health care expenditure costs and claims ratio (ratio of claims paid per revenue brought in). The claims ratio increased from 47% in 2012/13 to 63% in 2016/17 [10].

NHIF has 29 regional offices on the mainland plus an office in Zanzibar. Because of its size, the Dar es Salaam region includes three subregional offices. Regional managers are responsible for their districts down to the wards (subdistricts) for facility certification, reimbursement, advocacy, recruitment, and contributions and reporting to the national managers of each area who sit at the headquarters. Regional staff also oversee the contracts with employers and service providers as well as check claims to assure that provider services were rational.

Tanzania’s health financing strategy of 2017 includes plans to merge all the public prepayment schemes into a single national health insurance (SNHI) mechanism that would share a single risk and financing pool [10]. SNHI is intended to address the inefficiencies in Tanzania’s highly fragmented prepayment system and harmonize benefits, which is a critical step toward expansion [11, 12]. Legislation under a new SNHI plan will require mandatory enrollment for formal sector employees and will also use a tax increase and donor funds to make it financially viable [13, 14]. Mandatory enrollment is expected to deter the problem of adverse selection; however, members will still have different options from which to choose, and private health insurance will continue to be a complementary option. Whether or not retail outlets will continue to be covered as providers is unclear as the legislation is still being finalized.

## Overview of Retail Pharmaceutical Outlets

Tanzania has two levels of retail pharmaceutical outlets—full-service pharmacies and ADDOs or *Duka la Dawa Muhimu* (“essential drug shop” in Swahili). The country has 14,045 registered ADDOs throughout mostly rural and peri-urban areas and only 1,504 registered pharmacies, which are mainly clustered in towns and cities. ADDOs can sell over-the-counter medicines and a limited list of prescription medicines, such as commonly prescribed antibiotics. Stock outs in public facilities in the early 2000s prompted NHIF to start covering pharmacies as alternative providers. ADDOs were added to the coverage to address rural client demand, because 60% of civil servants are teachers who often live in rural areas with no pharmacies.

## Methodology

Two primary sources of data formed the basis for this case study: semi-structured in-person or phone interviews with key informants and government documents related to the country’s prepayment schemes. We purposively sampled government officials and conducted in-person interviews with 10 representatives from the NHIF (headquarters and Kinondoni zonal offices); the President’s Office of Regional Administration and Local Government, which oversees iCHF; the Pharmacy Council, which regulates the retail drug sector; and the district health offices of Bahi, Mvomero, and Gairo districts. Bahi is in Dodoma region, while Mvomero and Gairo are in the Morogoro region. The research team used convenience sampling to identify key informants from pharmacies and ADDOs. We conducted in-person interviews with owners or dispensers from seven pharmacies in the cities of Dar es Salaam, Dodoma, and Morogoro and an additional one pharmacy and one ADDO in the rural Gairo district. We conducted phone interviews with ADDO representatives in four additional districts and regions: Kakonko district (Kigoma region), Mkinga district (Tanga region), Chato district (Geita region), and Tuduru district (Ruvuma region). A total of 26 key informants were interviewed representing 18 distinct entities (Table 2). Our sampling was based on saturation principles, where we continuously recruited participants until we no longer received new information from their responses. We reference the sources of information as government, pharmacy, or ADDO representative, while maintaining their anonymity. Box 1 lists general topics we covered with both government and retail informants.

**Table 2:** Number of informants included in the study

		No. of Entities	No. of Informants
Government	NHIF	1	4
	President's Office of Regional Administration and Local Government	1	1
	Pharmacy Council	1	1
	District Health Offices	3	5
Retailers	Pharmacies	7	10
	ADDOs	5	5
Total number		18	26

Box 1: Selected topics of key informant interviews with government and retail representatives

- Level of knowledge in the community about NHIF

- Submitting and processing claims

- NHIF certification process and contract terms

- ADDO list of medicines

- NHIF reimbursement prices for medicines

- Current availability of medicines in health facilities and effect on retailer

- NHIF and access to medicines

- Challenges to an effective NHIF/retail outlet linkage

- How the NHIF/retail outlet linkage could be improved

Interviews at the participants’ workplace were conducted in English or Swahili or a mix, depending on their preference. Most interviews were recorded with notes taken, and interviews that were not recorded had a dedicated note taker. After reviewing the interview notes and recordings, government documents, and other publications, the research team triangulated responses among the various informants, and where possible, with information from the document review. The first author drafted a narrative on the perceived barriers and facilitators, which the rest of the research team reviewed for factual accuracy. In addition, we followed up with NHIF to answer specific questions to address gaps identified from the interviews.

The study protocol was approved by the Tanzania National Institute for Medical Research. Study participants were provided with information detailing the rationale for the study, their rights, and who will have access to information provided. All participants consented to participate in the study.

## Results

All of the pharmacies included in the sample had been in business for 5–19 years—two had started business as drug shops but had upgraded to full-service pharmacies. The number of NHIF clients served by pharmacies varied from 200 out of 3,000 total clients per month in Morogoro to 2,500 out of 5,000 total clients per month in a busy pharmacy on the outskirts of Dar es Salaam (P1, P2). The percentage of total sales that NHIF represents for pharmacies also varied, ranging from an estimated 15% to 50% of total sales (P1, P2, P3, P4, P5, P6).

The number of NHIF clients served by ADDOs in two districts was estimated to range from 10 to 15 clients per month (A3, A5). In terms of percentage of total sales, one ADDO in another district reported that 30% to 40% of sales came from NHIF, while another reported less than 5% of sales (A2, A5). An ADDO that has been NHIF-certified since 2004 estimated that up to 45% of sales used to come from NHIF clients, but that it had dropped to less than 10%, because a clinician from the health facility nearby had opened a pharmacy and was directing patients there (A4). The ADDO owner hoped to upgrade to a pharmacy to be able to better compete.

An NHIF informant confirmed that as of December 2019, NHIF had certified more than 7,000 health facilities including 200 accredited ADDOs (of 14,045 total) and 464 pharmacies (of 1,504 total), so clearly, retail outlets are a small proportion of their coverage. For instance, pharmacies and ADDOs received 8% and 0.01%, respectively, of total benefits payments by NHIF for the period July to September 2018 [G3]. NHIF records indicated that only 28 ADDOs received reimbursement in the 2018/19 fiscal year for about TSH 25 M. This compares with reimbursement of almost TSH 200 M in 2015/16, before the beginning of a steady decline. In comparison, NHIF reimbursed pharmacies more than TSH 32B in 2018/19.

All respondents felt that NHIF certification helped their business and clients to some degree. Two pharmacies mentioned that membership allowed NHIF clients to afford more expensive drugs and to buy the full course as opposed to cash clients, who sometimes only buy half-doses (P6, P4). Two retailers noted that it was guaranteed payment for them (P2,A2). However, as outlined below, perceptions regarding the barriers and facilitators of engagement varied between the government officials and retailers.

### NHIF-retail outlet certification process

The NHIF has a standard inspection checklist for retailers. Key criteria include registration by the Pharmacy Council, valid business permit, and taxes paid. Several retailers thought that having a computer and internet connection was a requirement for certification (P5, P7, A1), and an ADDO informant said that failure to meet this requirement led to their NHIF certification not being renewed (A1). However, one NHIF informant stated that it was not a requirement and observed that many certified health facilities did not have computers (G4). Once a retailer applies for certification and the facility has been inspected, the NHIF accreditation committee makes the final decision. The application fee is TSH 20,000 and the certification fee is TSH 200,000. The contract issued by NHIF, which lasts for 3 years, defines what is covered, payment terms, and termination procedures.

The NHIF zonal representative estimated that their office received five to six applications per quarter and that the dropout rate in their area was minimal, which they cited as an indication that retail providers really want to work with the NHIF (G2). NHIF sends a letter 3 months before a contract expires to inform providers that they need to re-apply (G2). However, the process for contract renewal has been problematic for at least one retailer, who complained about the lack of communication and described their experience this way:

“Communication is a big problem. [We] requested an extension three months in advance, but it took time, and it expired. NHIF [said] to go ahead and continue with service, but then the system kicked [us] out. Those claims took five months to pay.” —Pharmacy administrator (P2)

That experience is contrary to those of several other pharmacy informants who described the renewal process as being fairly straightforward (P4, P5, P6, P7). One pharmacy informant who was on his third NHIF contract assumed that recertification was dependent on previous performance (P5).

According to one NHIF informant, the Fund certifies according to geographic need, but retailers have to take the initiative to apply to avoid conflicts of interest (G2). However, several informants from pharmacies and ADDOs claimed that NHIF had contacted them about becoming certified; however, that contact appeared to have been more than 10 years ago (P4, A2, A4). One government official noted that there was less of a need for NHIF-certified private sector facilities, because the government was pushing for pharmacies to open in public hospitals using revolving drug funds (G7). In one district, the hospital had opened its own pharmacy using a revolving drug fund and was planning to link it to the NHIF. The district official outlined plans to do the same at the other health centers and observed that so far, prices were a bit cheaper than in retail outlets (G11).

### NHIF-covered medicines and pricing

From 2010 to 2018, medicines and health supplies made up on average, 43% of NHIF’s claim reimbursement [6]. Generally, NHIF automatically covers medicines on the national essential medicines list and does not cover branded medicines (G1). The NHIF sets reimbursement prices based on a market survey; wholesale, manufacturer, and Medical Stores Department prices; inflation and margins; and consultations with stakeholders including representatives from health facilities and the retail sector [10] (G1). This process is supposed to be carried out every 3 years according to NHIF but since 2001, the price list has been updated in fiscal year 2007/08, 2012/13, and 2016/17 [10]. NHIF said that individual prices can be amended during the 3 years, but there is no indication that it happens routinely. NHIF reimburses health facilities and retail outlets at the same price for medicines and health supplies.

The NHIF central-level representative claimed that the agency was not receiving complaints about price reimbursement for medicines (G1); however, several pharmacy informants complained that they sold medicines at a loss, because rates were sometimes lower than their cost for the medicines (P1, P2, P3). One informant noted that they refused to fill the prescription for such products and complained that there is no regional variation in reimbursement rates, which was problematic, because being located outside of Dar es Salaam increases transport costs and wholesaler prices (P1). Another pharmacy informant believed the price list had not changed since 2016 and highlighted the fact that the pharmacy was contractually obligated to sell the medicines no matter what price the pharmacy had paid, even if it meant losing money (P2). At least one district official agreed with the retailers, claiming that reimbursements were not based on market prices and needed to be updated more often (G8).

### NHIF claims process

Retailers were generally happy with the speed of reimbursements but claim rejection was by far the most contentious topic among those interviewed. The primary issue with claims centered around prescriber error or discrepancies between electronic and written prescriptions. This was despite the NHIF’s provision of office-based claim training for providers, supportive supervision of the claims process, and a help number for providers to call. [G2].

#### Reimbursement process

The pharmacies’ process for reimbursement involves filling out a form with copies of the prescriptions and either submitting the paperwork electronically (P2) or taking it physically to the NHIF zonal office (P1, P3). Most retail outlets said they submitted their claims every month or two depending on how many claims they have. Contractually, NHIF is required to pay within 60 days, and retailers generally agreed that reimbursement came within 2–3 months. All of the ADDOs except for one said they received reimbursement within 1–2 weeks; one said it took 3 weeks or more. The outlets receive the reimbursements directly into their bank accounts, and most acknowledged that the speed of reimbursement was not a problem, although one pharmacy noted their June 2019 submission had not yet been paid in December 2019 (P6). Payments are processed regionally, so performance varies across the regions (G2). The NHIF zonal office was working to reimburse outlets within 15 days as a goal. Multiple pharmacy and ADDO respondents commented that the reimbursement process had improved immensely.

“The reimbursement system now is very encouraging compared to how it was in the past. We used to have outstanding claims, say, for about seven months but now we are paid within the two months, which are in the contract.”—Pharmacy owner (P4)

#### Claim rejections and deductions

NHIF claim rejections occur if a prescription lacks information such as a date or prescriber signature or does not conform to national standard treatment guidelines (STGs). Contractually, retail outlets and health facilities can only be reimbursed for prescriptions that follow the STGs. This means the right medicine for the diagnosis and the medicines being prescribed at the appropriate health facility level. Therefore, if a prescription for treating a simple condition is coming from a hospital rather than a primary health care center without a referral, the NHIF rejects it. It is up to the dispenser to know what the STGs indicate as appropriate treatment, carefully examine each prescription, and send the patient back to the health facility if there are problems. As one informant outlined:

“Deduction of reimbursement for improperly filled prescriptions, although in some cases they are fair, sometimes they are unnecessary. Before issuing medicines, we must ensure that every part of the prescription is filled properly and that medications are correct and in line with STG and NHIF list and that the dosage and duration is correct. […] If we issue medicine from a prescription with gaps, NHIF deducts the payments either the whole prescription of some of the medicines within the prescription.” —Pharmacy owner (P4)

In addition to providing patients with a written prescription, in computerized health facilities, prescribers file the prescription electronically. When the patient goes to fill the prescription, the pharmacy employee reconciles the written prescription from the client with the electronic copy. If the two prescriptions match, then the prescription can be filled. The reconciliation of the electronic and paper prescription is NHIF’s mechanism to combat fraud. (ADDOs use a completely paper-based process that does not include the two-prescription reconciliation, and not all health facilities have access to computers or the internet.) The electronic–paper verification of prescriptions has proven problematic for both the pharmacies and their clients. If the prescriber has not entered the prescription into the electronic system when the client submits the paper prescription to the pharmacy, or if there are discrepancies between the two versions of the prescription (for example, the date is wrong on one), the pharmacy cannot make any changes to the paper prescription and often has no choice but to return the customer to the facility to resolve the problem. One pharmacy estimated that 20% of their NHIF clients had prescriptions with discrepancies and had to be sent back to the health facility (P2), which “causes a disturbance to the clients.” Another informant observed:

“Returning clients to the facility because the prescription is incomplete or inaccurate is becoming a challenge here because we do return many patients to facility. We also feel bad and sometimes we fear to tell the patients because we also feel the inconvenience—imagine you have to return someone to the hospital? But there is nothing we can do.”—Pharmacy owner (P4)

Pharmacists believe they are frequently penalized for poor prescribing practices by the NHIF, which rejects their reimbursement claims (P1, P3, P4, P5). One pharmacy owner estimated that TSH 8 M worth of claims on a total of TSH 82 M—or 10%—had been rejected in the previous month and felt that NHIF should deal directly with the prescribers when there is a problem rather than just rejecting the pharmacy’s claim out of hand (P1). Another estimated that deductions at his pharmacy were TSH 300,000 to TSH 1 M per month and bemoaned the challenge:

“[We are] providing a service to the community and to NHIF, but they are exploiting mistakes. NHIF needs to be more flexible. […] If no changes happen on the deductions, we will pull out. It’s the prescribers’ mistake that the pharmacies pay. […] If they can fix the deduction, everyone will enjoy NHIF.”—Pharmacy owner (P3)

Almost every informant we interviewed from NHIF, districts, and retailers agreed that the quality of prescriptions coming from health facilities was the biggest problem. One NHIF informant acknowledged that the Ministry of Health, Community Development, Gender, Elderly and Children (MOHCDGEC) should be monitoring prescribing practices at facilities, but that quality assurance and supervisory practices were lacking (G2). And although these problems lie with the prescriber or facility, as the interface between NHIF and the client, the pharmacy staff often receive the brunt of the clients’ frustration. These issues have created barriers to access to medicines for some NHIF members. Some members pay for the medicine out-of-pocket or go without rather than go back to the health facility to correct the prescription (P2).

Although the pharmacy owners’ perception was of significant losses in rejected claims, NHIF data from the last 10 years showed that the average claim reimbursement rate was 98% for pharmacies and 95% for ADDOs (G3); however, interestingly, none of the ADDOs we interviewed commented on having problems with deductions. Perhaps their perception was different because they were not subject to the paper/online prescription reconciliation process, which can be a frequent headache for pharmacies. On the other hand, ADDO dispensers may not be as educated as their pharmacy counterparts on practices, and they do not have access to online or other resources to check against the STGs, for example. Therefore, if they fill a problem prescription, NHIF will reject it, and the ADDO will not get reimbursed.

#### Contesting claims

As for retailers’ ability to contest NHIF decisions regarding rejected claims, the NHIF representative said that claims can be contested for 6 months after filing and that they would contact retailers if they saw an issue with a claim that came in. If it was a problem on the NHIF side, they would pay it; many retailers, on the other hand, seemed to feel that fighting a decision on a claim was useless. However, NHIF believed that it was more a problem that the pharmacy dispensers were underqualified, so prone to error. Maintaining quality is a priority for the NHIF—no pharmacy should dispense a prescription if it does not follow the STGs. The NHIF representative noted that dispensers should contact their zonal office if they had any questions about the prescription, and that the office spent time training both pharmacy dispensers and prescribers because they wanted to minimize these problems. Although district pharmacists oversee pharmacy and ADDO operations, their involvement with NHIF in their districts varies. Two district pharmacists said they tried to mentor pharmacies on the NHIF paperwork and facilitated interaction with NHIF in the case of problems, while the other said she had no involvement in the process.

#### Fraud

NHIF stated that fraud was always a problem with all service providers, not just the retail sector (G1, G2). An example would be pharmacies that team up with public health facilities or are owned by a health facility staffer, which facilitates fraudulent activities, such as repeated referrals from a health facility to the same pharmacy for out-of-stock items. NHIF uses the online system to scan for over-prescription and excessive referrals to the same outlet. They sometimes follow up with patients to see if they actually received the medicine that was supposedly prescribed. If there is suspicion about a facility or pharmacy, NHIF performs an inspection. They can de-certify the facility and deduct funds. Ongoing inspections are risk-based, and inspections occur less frequently after initial certification (G1). Informants from the pharmacies agreed that inspections occurred only if there was a problem (P1, P4). However, the ADDO informants noted that their districts only checked to see that they were registered by the Pharmacy Council.

### Issues Specific to ADDOs

Tanzania’s Health Sector Strategic Plan IV says that the MOHCDGEC would “encourage the ADDOs to engage in greater self-regulation and build their own capacity so that there is greater access to approved medical products, especially in rural areas.”[14] However, because ADDOs are not allowed to sell a wide range of prescription products, they can only supplement a limited number of public facility services. Health facilities generally keep common drugs in stock, which overlap with what ADDOs are allowed to sell, but there is a gap with less common drugs that are not on the ADDO list (A3). All of the ADDO representatives and many respondents from the other sectors said that limitations to the list was a barrier to ensuring community access to medicines—the NHIF zonal representative recommended including more medicines for chronic conditions on the ADDO list in line with Tanzania’s changing epidemiology. One ADDO owner said that clients complained about not being able to get medicines either at the facility because of stock-outs or at the ADDO, because they were not on the approved list (A1). In addition, because the ADDO list has not been updated since 2015, it no longer aligns with the STGs, which complicates NHIF reimbursement. Two ADDO informants mentioned that the ADDO list included products that are no longer prescribed, such as procaine penicillin fortified (A4, A5).

“With the list we have, I am not sure if ADDOs can continue to provide NHIF services—maybe in very remote areas where health facilities are far and [patients] need common drugs available at ADDO level.”—ADDO owner (A5)

A district official felt that the reimbursement price list would need to be updated because ADDOs make so little profit on inexpensive essential medicines, making claim rejection too much of a financial risk (G9). A pharmacy, which had previously been an ADDO, agreed with that assessment (P7).

One district official was unaware that NHIF accepted ADDOs as providers and thought that ADDOs also needed to be sensitized on how to apply to become NHIF-certified (G12). The official thought that NHIF members in the district would use ADDOs if they had that option, even with the limited list, because the one pharmacy in the district was not reliably open. Another district official stated that citizens in her district had to travel 100 km to another town to access the closest NHIF-certified outlet (G10).

Compounding the situation in rural areas, we learned from our district government informants that paying for NHIF coverage was often beyond the means of their citizens, and iCHF did not cover ADDO purchases. One central government respondent noted that if iCHF oversight was decentralized to the district councils, then they could choose to link with ADDOs in their community. Another suggested that local government contracts with ADDOs would be a potential option, but that iCHF’s reimbursement rates would not be able to compete with those of NHIF because of NHIF’s greater resources. Several district informants also felt that a linkage between iCHF and ADDOs would make iCHF more attractive for members.

### Community knowledge about NHIF

Most of the informants interviewed agreed that community knowledge of iCHF was better than knowledge of NHIF. This is not surprising, because in addition to being heavily promoted, iCHF has community enrollment agents. NHIF is using social media and media tours—radio, flyers, community outreach—as part of its *vifurushi* expansion. One retailer felt that most people who were not civil servants were unfamiliar with NHIF but that familiarity was increasing (P4):

“When patients come to pharmacies to buy medicines which are costly while they see others getting for free with NHIF membership, they start enquiring on how to join. When they ask NHIF they realize that it is only meant for some people then they were getting stuck but now with the new packages then we hope that most people will now join.”—Pharmacy owner (P4)

One district official suggested that village meetings should be used to increase knowledge of the NHIF *vifurushi* (G9). However, the fact that this official was also unfamiliar with the packages suggests that some district officials may themselves need education.

Despite the apparent knowledge increase among communities regarding NHIF, beneficiaries were in some cases unaware of what is in their benefits packages, especially when there had been changes. One district official observed that NHIF members were not informed about the details of their coverage and has tried to fill this gap by handing out information flyers to inform beneficiaries, for example, of what to do if their card is lost (G11). Some retailers also complained that NHIF members were not well informed about their coverage and attributed it to the NHIF being insufficiently transparent about changes to benefits packages (P2, P3, P6). Clients had been blaming pharmacies for not covering medicines, and pharmacy staff have had to explain benefits changes to the clients (P1, P2, P6, P7). Clients were often upset and confused when medicines that they previously received as part of their NHIF benefit were suddenly no longer covered without warning or explanation, “It’s a pinch for customers” (P2). Another further noted:

“NHIF should educate members about the new list of medicines—members aren’t given information about coverage. People think it’s the problem of the pharmacy and not the list, so they blame the pharmacy and tell their friends that this pharmacy doesn’t sell NHIF drugs […] They don’t understand that the problem is a change in coverage and not the pharmacy denying the coverage.”—Pharmacy employee (P1).

A pharmacy employee noted that pharmacies often did not know about changes until a claim was denied (P2). Two respondents recommended that NHIF put the drug list online to make it more transparent to NHIF members, retail outlets, and to the health facility staff (P1, P2).

### Access to medicines

When we asked respondents if they thought the NHIF in general, and the linkage with retail outlets specifically, had increased access to medicines, answers were generally positive from both government and outlet respondents. For example, three district health officers thought that the NHIF and iCHF helped increase access to medicines in health facilities, because a proportion of member fees went toward procurement of pharmaceutical products—67% of the fees in Gairo (G8,G9,G11). The retailers focused on how they provided access in cases where the health facilities are out-of-stock, and a district official complained that because his district only had one pharmacy and few ADDOs that when health facilities ran out of medicines, there was no back-up (G11). NHIF members visiting a new pharmacy that was awaiting NHIF certification were disappointed to find out that the pharmacy was not able to serve them when the health facility is stocked out (P7).

“NHIF have significantly increased access to medicines because in the past there were medicines which could not be easily accessed by clients because of high cost but now they can access them in full dose because the cost has been covered by NHIF.” — Pharmacy owner (P4)

The same owner also commented that they now stock products that they did not previously stock, because NHIF now covers them. Another said that NHIF patients could access expensive medicines, multiple medicines, and sufficient quantities of medicines, especially those with chronic illnesses such as diabetes and hypertension (P6).

When we asked pharmacies if better availability of pharmaceuticals in health facilities affected their business, they said no. One surmised that only the availability of common essential drugs had increased in facilities, which did not include expensive medicines or those used for chronic diseases, such as antihypertensives (P6). Unlike the pharmacies, ADDO informants felt their number of clients depended highly on the availability of medicines in the health facilities. An ADDO owner stated that ADDOs saw few NHIF clients compared to pharmacies, because ADDOs had such a restricted list of medicines allowed for sale (A1). Our ADDO respondents all thought that pharmaceutical availability in health facilities had increased, but that fluctuations in stock still occurred, especially at the end of the month, which affected the number of customers they see.

## Discussion

The findings of this study point to several facilitators and barriers for NHIF engagement with private retailers. Important enablers include widespread awareness of the insurance scheme, the seemingly straightforward accreditation process, and the speed of reimbursements. Retailers were all aware of the NHIF and saw value in being a certified provider for their own business interests and for the benefit of their clients with respect to increased access to medicines. Awareness of NHIF, although still less than that of iCHF, also extended into the community, with retailers recounting instances of clients enquiring about the NHIF after learning about its benefits from other clients. Customer demand increases engagement as the higher the proportion of clients enrolled in the scheme, the higher the financial incentive for retailers to participate in NHIF [3, 15]. Despite some confusion regarding the requirement for computers and internet, the retail informants in general described a straightforward accreditation and contract renewal process. Complexity and ambiguity of the accreditation process have been identified as a barrier in other contexts [3]. Retailers were pleased with the speed of reimbursements and pointed to positive changes over the years. Although reliability or speed of reimbursements was positively linked to retailer participation in an insurance scheme [16], in Tanzania, this may be diminished by dissatisfaction with the reimbursement prices, which several informants felt were below the market, and with problems with rejected claims. The retailers’ ability to cover service costs was an important factor in their willingness to participate in the insurance scheme [16].

ADDO owners believed that client demand for medicines was associated with the availability of medicines in public health facilities. This suggests that the ADDOs are functioning as a ‘stop gap’ for the public sector. However, the misalignment between patient needs and the medicines approved for sale at the ADDOs has the potential to limit access, particularly in rural areas. One potential solution is to expand or at least update the list of medicines that ADDOs can sell. The responsibility for ADDO operations, however, is fragmented: the Tanzania Medicines and Medical Devices Authority is responsible for keeping the ADDO list of medicines updated; the Pharmacy Council oversees accreditation of premises and personnel; and NHIF coverage is limited to what the ADDOs are legally allowed to sell, although that list has not been updated since 2015. Bringing together these stakeholders around a common agenda of updating the list could increase pharmaceutical access for NHIF members, particularly in rural areas that lack full-service pharmacies. And given that 200 ADDOs are NHIF-certified, but only 28 filed claims in 2018/19 likely indicates that a combination of supply and demand factors contributes to underutilization. In addition, a stronger information linkage between the Pharmacy Council and NHIF related to NHIF certification status or fraud, for example, would increase efficiencies.

Other potential barriers to NHIF engaging private retailers include poor communication, poor prescribing practices, fraud prevention mechanism, claim rejections, and NHIF member dissatisfaction. The findings suggest an interplay between all these factors, particularly for pharmacies—poor communication by NHIF with its members and providers coupled with poor prescribing practices result in NHIF member dissatisfaction with retailers when they are denied service and revenue loss for retailers from claim rejections. The retailers serve as the primary interface between patients and the NHIF with respect to their medicines benefit, so often serve as the main target for customer dissatisfaction for issues beyond the retailers’ control: first, NHIF does not properly communicate benefits changes with its customers, which creates confusion and places pharmacies in the position of having to explain changes to customers or turn away customers who may be unable to afford out-of-pocket payments. Second, when prescribers fail to follow STGs or fill out prescriptions accurately, members may have to either return to the facility to correct the problem or pay out-of-pocket or go without their medicine. These prescribing issues can also result in loss of revenue for the pharmacies, who feel like they are routinely penalized by NHIF for prescribers’ errors.

Some of these perceived barriers could be mitigated by improved communication between the NHIF, its members, and retailers. Communication likely varies by district, depending on the responsiveness of the NHIF focal person at district level and the staff at the appropriate zonal office who process claims. One informant suggested bringing together NHIF and pharmacies and other stakeholders to discuss challenges on a bimonthly schedule, particularly related to claim rejections (P1). Another suggested that NHIF place dedicated staff in major health facilities to help address these challenges and provide a resource to patients to minimize their back-and-forth trips to pharmacies (P6). Another potential mitigation is for the MOHCDGEC to concentrate on improving prescribing practices to minimize problems for pharmacies and ADDOs and particularly for NHIF members who often have to make return trips to health facilities to rectify prescription problems.

## Limitations

This study has several limitations. We used convenience sampling for our interviews, so there may be underrepresentation in the types of outlets and their distribution between rural and urban areas. We mitigated for some of this by supplementing our face-to face interviews with telephone interviews. However, the results cannot be generalized to NHIF-retail outlet relationships in other geographical areas or strata, such as urban versus rural locations. The client perspective is missing from our findings. Due to resource constraints, we were unable to include clients as informants. As such, we could not triangulate information regarding client experience and satisfaction with NHIF and access to coverage through the retail outlets. Our study may be, therefore, biased toward the NHIF and provider perspective.

## Conclusion

The Tanzania Health Sector Strategic Plan IV noted that public health facilities chronically lack essential medicines and that the trend for improvement was not promising [14]. A recent government audit of primary health care facilities showed that only 11 of 101 sampled facilities had 10 essential tracer items available during 12 months of 2019 [17]. Based on this less-than-optimistic government view and the audit results, expanding access to medicines through an alternative to public sector facilities should be a priority. In addition, Suchman and colleagues [18] pointed out that for countries to achieve UHC, private providers must be included in health insurance schemes; they added,

“Since private providers rarely interact with government systems to the extent that public providers do, it is challenging for them to give [social health insurance] officials the meaningful feedback that could result in these systems becoming friendlier to the private sector. Greater formal collaboration between the public and private health sectors is an obvious way to facilitate this feedback and, as a result, strengthen health systems.” [18 p. 778]

Although the level of knowledge and communication among our respondents about the relationship between NHIF and retail drug outlets varied, the retail respondents were generally positive about being NHIF-certified, and several commented that NHIF services had significantly improved since earlier years. Putting into place a mechanism to increase feedback between NHIF and the private sector and promoting the government’s consideration of how ADDOs can better serve their rural clients can help increase access to medicines as part of Tanzania’s progression toward UHC.

## Data Availability

Copies of the interview questions and notes are available from the corresponding author on reasonable request.

## Abbreviations

ADDO: Accredited drug dispensing outlet
CHF: Community Health Fund
iCHF: Improved Community Health Fund
MOHCDGEC: Ministry of Health, Community Development, Gender, Elderly and Children
NHIF: National Health Insurance Fund
SNHI: Single national health insurance
STGs: Standard treatment guidelines
TIKA: Tiba Kwa Kadi
TSH: Tanzanian shilling
UHC: Universal health coverage
